# Remarks on Pneumonia; Being an Abstract from a Report of Cases Treated at Fort-Pitt Hospital

**Published:** 1823-05

**Authors:** James Forbes

**Affiliations:** Deputy Inspector of Military Hospitals.


ART. II.—*Remarks on Pneumonia; being an Abstract from a Report of Cases treated at Fort-Pitt Hospital.* By JAMES FORBES, M.D. Deputy Inspector of Military Hospitals.

DISEASES of the order Phlegmasiæ, affecting the respiratory organs, may be arranged under three heads:—1. Primary acute inflammation; 2. Acute inflammation supervening upon chronic; 3. Chronic inflammatory action succeeding to acute.

Dr. Cullen has, in his Nosology, attempted to establish a diagnosis between inflammation of the parenchyma and investing membrane of the lungs, and Dr. Good has followed his example; yet, with that good sense which characterized him when really practical points were in question, he says, "Neither do our diagnostics serve to ascertain exactly the seat of the disease; nor does the difference in the seat of the disease exhibit any considerable variation in the state of the symptoms, nor lead to any difference in the method of cure:" and again, "It seems to me very doubtful if any acute inflammation of the lungs, (i. e. the case of an inflammation beginning in the paranchymatous or cellular texture of the lungs, and having its seat chiefly there,) or any disease which has been called Peripneumony, be of that kind." Inflammation of the substance of the lungs, as a primary affection, may therefore be presumed to be very rare, excepting in cases where these organs are in a state of excitement from other causes, or of disorganization. If it does occur, it is commonly communicated, more or less, to the investing membrane. This membrane may be considered as, in general, the seat of active inflammation. I am surprised to find Dr. Good stating, under the head of Pleuritis Vera, that the inflammation, in this case, commences on that side of the membrane which lines the ribs; while it is distinctly, and I believe truly, stated by Dr. Cullen that this is a rare occurrence, and that the disease much more frequently begins in, and chiefly affects, the pleura investing the lungs. The inflammation of that portion of the pleura which covers the upper surface of the diaphragm, has been distinguished by a particular name; and Dr. Good states, that "painful constriction around the præcordia, and small quick pulse, and laborious breathing," sufficiently decide that the inflammation is seated chiefly in the diaphragm; abandoning the older diagnostics of phrenitic delirium, risus sardonicus, and other convulsive motions, with which this variety of thoracic inflammation was supposed to be attended. Dr. Darwin says he could distinguish paraphrenitis, or pleuritis diaphragmatica (as it is more correctly denominated by Good), from pleuritis, by observing whether the patient carried on respiration most by the elevation of the ribs, or the depression of the diaphragm. In pleuritis, he says, the ribs remain as much as possible at rest, and respiration is carried on by depressing the diaphragm; while, in paraphrenitis, on the contrary, the elevation and depression of the ribs goes on alternately, but the diaphragm remains at rest, and no movement of the abdomen is perceptible.

It is fortunate that the practice is not influenced by these ingenious divisions, with their accompanying diagnostics; for they certainly do not serve to ascertain the seat of disease in a satisfactory manner. It is extremely proper, however, to attend to the mode in which respiration is performed in all inflammatory affections of the thoracic, and also of the superior abdominal viscera, particularly in hepatitis, where the patient avoids as much as possible depressing the diaphragm. Structure, nature of function, and vicinity to the heart, render the respiratory organs particularly liable to disease; and the same circumstances would lead us to presume that inflammation of those organs would make rapid progress, and, when not speedily arrested by the power of medicine, terminate shortly in an unfavourable manner. We are often surprised, however, to observe cases in which acute inflammation has gone on unmolested for eight or ten days, and even longer, and has yet terminated happily at last. Such protracted cases frequently occur here in men who have been attacked on a march, and who make great efforts to reach the hospital. Although symptoms of effusion having taken place in many of them occurred, and they continued to labour under cough and dyspnœa for a considerable time, yet they all ultimately recovered. Indeed, of ninety-two cases treated in all stages during the last twelve months, not one has proved fatal; for, although seven of them be reported as such, they were in reality cases of genuine phthisis pulmonalis, as was afterwards ascertained by dissection.

I have, on former occasions, strongly urged the necessity of copious blood-letting in this disease, and stated how far preferable one full bleeding is to small and repeated evacuations, even although amounting in the aggregate to a greater abstraction. I have now only to state, that, when there is not something very striking to contra-indicate it, the blood is permitted to flow *pleno rivo*, until respiration is performed without pain, and the pulse reduced in force and frequency. Should syncope occur previous to these effects being produced, the flow of blood is stopped until the patient recovers, and is then permitted to go on again; and I have seldom known any tendency to syncope recur. The generality of patients have lost from thirty to forty ounces of blood at the first bleeding, many from fifty to sixty, and some have borne with advantage the loss of from sixty to seventy ounces. It is true that many patients, from age, constitution, or other circumstance, cannot bear such copious depletion; but these are comparatively few, and the generality of cases admitted into this hospital, though with broken-down constitutions, bear with ease and advantage full (and, if necessary, repeated) bleeding. The advice of various authors to bleed until the buffy coat disappears, must be taken with a few grains of allowance; yet its presence or absence is by no means to be neglected. Many circumstances in blood-letting, it is true, may prevent the formation of the buffy coat in blood otherwise disposed to it, so that we cannot conclude against the presence of inflammation from its absence; but, in almost all cases in which the blood does exhibit this appearance, joined with other symptoms, we may with tolerable certainty infer the existence of inflammatory action. A frequent pulse is by no means an invariable symptom of pneumonic inflammation; for several severe cases occurred where it was scarcely at all accelerated. A slow pulse ought not, therefore, to deter us from employing the same active means as we would do in cases where it is frequent. Blisters and digitalis are no doubt useful occasionally, when large bleedings are contra-indicated; but, in general, I have little faith in them. The most useful auxiliary to blood-letting, in diminishing the action of the heart and arteries, is the tartarized antimony, given in nauseating doses. The objections against its use in fevers do not apply here, as the stomach is seldom in an irritable state in pneumonia; and I can say, from experience, that, with a little attention to regulating the dose, its effects will be highly beneficial. In protracted cases, where symptoms of effusion had taken place, a combination of squills, digitalis, and calomel, was generally and successfully employed.

CASE I.—Edward Dougherty, ætat. twenty-five, (admitted into the hospital, January 3,) complains of acute pain in the left side of the thorax, much aggravated by a full inspiration; breathing much oppressed, with a dry troublesome cough; pulse 110, full; tongue furred; skin hot; thirst very urgent. Has alternate rigors and accessions of heat; was attacked with these symptoms ten days ago.—Mittatur sanguis ad deliquium detrahendo ℥lviii. et admoveatur Emplast. Lyttæ parti affectæ; nec non habeat ʒvj. Magnesiæ Sulph.

4th.—Pain and difficulty of breathing much alleviated; cough abated in frequency; pulse 108, softer; tongue white; thirst urgent.—Rept. haustus Cathart.

5th.—Pain of chest gone; breathing performed with perfect freedom; pulse 96. Appetite returning.

This patient was transferred to the convalescent hospital on the 10th, free from all complaint.

CASE II.—Joseph Morgan, ætat. fifty-one, (admitted into the hospital January 21,) complains of acute pain in the chest beneath the sternum, difficulty of breathing, and severe dry cough. Pulse 102, hard; tongue white. The above symptoms have been present nine days. Has been twenty-eight years in India, during which time he enjoyed an uninterrupted state of good health.—Detrahatur sanguis e brachio ad ℥xlv. et admoveatur Emplast. Lyttæ parti dolenti. Capeat statim ℥ss. Magnesiæ Sulphatis in Infusi Sennæ, ℥ij. solutæ.

22d.—Pain of chest gone; breathing performed with freedom; cough less urgent; pulse 92, soft; bowels open.

This patient had cough for some days after. He was transferred to the convalescent hospital on the 5th of February, free from complaint.

CASE III.—Donald Mohagan, ætat. twenty-three, (admitted Feb. 1st,) labours under acute pain in the left side of the thorax, difficulty of breathing, and frequent cough, with scanty expectoration. Pulse 110, and hard; tongue furred. These symptoms came on five days ago; but he has been affected with cough and hurried respiration, on using exercise, for about a year.—Detrahatur sanguis e brachio ad ℥xlvj. et capeat ℥j. Magnesiæ Sulphatis ex aquæ cyatho.

*Vespere.*—Pain continues in the left side; breathing much impeded; pulse 108; bowels open.—Rep. detractio sanguinis ad ℥xxx. et admoveatur Emplast. Lyttæ parti dotenti.

2d.—Pain of side removed; breathing performed with more freedom; pulse 102, and soft; cough continues.—℞. Antimon. Tartar. gr. ij.; Liq. Ammon. Acet. ℥iij.; Infusi Lini, ℥v. M. Capeat cochleare omni hora.

3d.—Cough was very urgent last night, and produced considerable pain of the head. Pulse 98, full and hard; bowels open; breathing impeded.—Rep. detractio sanguinis ad ℥xxx.

He was considerably relieved by the bleeding, and he continued to mend during two days, at the expiration of which the cough and dyspnœa became urgent, attended with frequent, hard pulse. In consequence of this he was again bled to ℥xxx. by which the symptoms were considerably relieved: he, however, continued to be affected with cough and dyspnœa, on using exercise, for several months afterwards.

It will be seen that this patient lost 136 ounces of blood in five days; seventy-six ounces having been abstracted during the first day.—We could adduce more cases in which depletion was carried to the like extent, but this one may suffice as an illustration of the extent to which the depletory system may be carried.

CASE IV.—James Taylor, ætat. thirty-five, (admitted into the hospital, February 11th,) complains of acute pain about the middle of the sternum, difficulty of breathing, and alternate rigors and accessions of heat; urgent thirst; and frequent severe cough, with scanty expectoration. Pulse 110, full and hard; tongue furred. He has had cough for more than a fortnight, but the above acute symptoms came on three days ago.—Detrahatur sanguis e brachio ad ℥lij. et capiat ℥j. Sulphatis Magnesiæ, nec non admoveatur Emplast. Lyttæ sterno.

12th.—Pain gone; breathing performed with freedom; pulse 100, and soft; cough continues. Bowels freely opened.

The cough continued some days, when it entirely left him; and he was discharged on the 24th, free from complaint.

(Various other cases are contained in the Report; but those we have given will be sufficient to illustrate the practice recommended. In an ensuing Number, we will lay before our readers the observations of Dr. FORBES on Acute Bronchitis. —EDITORS.)

